# Prognostic value of integrin variants and expression in post-operative patients with HBV-related hepatocellular carcinoma

**DOI:** 10.18632/oncotarget.20161

**Published:** 2017-08-10

**Authors:** Liming Shang, Xinping Ye, Guangzhi Zhu, Hao Su, Zhixiong Su, Bin Chen, Kaiyin Xiao, Lequn Li, Minhao Peng, Tao Peng

**Affiliations:** ^1^ Department of Hepatobiliary Surgery, The First Affiliated Hospital of Guangxi Medical University, Nanning, China; ^2^ Department of Hepatobiliary Surgery, Affiliated Tumor Hospital of Guangxi Medical University, Nanning, China

**Keywords:** integrin, SNPs, *ITGA5*, *ITGB5*, hepatocellular carcinoma

## Abstract

Integrins are a large family of cell surface receptors that bind extracellular matrix proteins and participate in cancer progression. However, the prognostic value of integrin family genes in post-operative patients with HBV-related hepatocellular carcinoma (HCC) remains unknown. In this study, we investigated 18 single nucleotide polymorphisms (SNPs) in integrin family genes and found that the AG/GG genotypes at rs988574 in *ITGA1* predicted a better prognosis compared to carriers of the AA genotype (*P* = 0.025, HR = 0.69, 95%CI = 0.50–0.96). Moreover, rs988574 genotype combined with serum level of AFP had a better prognostic value in HBV-related HCC patients (*P* = 0.026, HR = 1.75, 95% CI = 1.07–2.85). Furthermore, we compared the expression of 24 integrin family genes in HBV-related HCC tissues and adjacent normal tissues. Survival analysis demonstrated that expression of three of the family members, *ITGA5*, *ITGB5* and *ITGA2B*, were significantly associated with the overall survival (OS) or relapse-free survival (RFS) of HBV-related HCC patients. Additionally, patients with lower expression of both *ITGA5* and *ITGB5* had the best OS and RFS (*P* = 0.017 and *P* = 0.002, respectively). Our study demonstrated that rs988574 of *ITGA1* and the expression of *ITGA5*, *ITGB5* and *ITGA2B* are potential independent prognostic bio-markers and therapeutic targets for HBV-related HCC patients and may be useful for the diagnosis of HBV-related HCC.

## INTRODUCTION

Hepatocellular carcinoma (HCC) is the second leading cause of cancer-related death worldwide [1]. More than 500,000 patients are newly diagnosed with HCC every year, and 50% of all cases and deaths are in China [2, 3]. Among these, most are hepatitis B virus (HBV)-related HCC patients. Although advances in treatment, especially in surgical techniques and molecular targeted therapy, have improved the survival rates of HCC patients, the long term prognosis after surgical resection remains very poor [4–6]. Thus, early diagnosis and treatment are very important to improve the prognosis of HCC patients [7–10]. Multiple clinical factors have been used as indicators for the diagnosis and evaluation of HCC, for instance, drinking status, chronic HBV or hepatitis C virus (HCV) infections, large tumor size, vascular invasion, positive portal vein thrombosis, serum alpha fetoprotein (AFP) and Barcelona Clinic Liver Cancer (BCLC) stage [11–13]. Current indicators used to predict HCC outcomes and the prognosis of HCC patients are not sufficient. Thus, it is urgent to identify potential biomarkers for improving the efficacy of prognosis prediction and the clinical outcomes of HCC patients.

The integrin family consists of 18 α and 8 β subunits that form 24 known αβ-heterodimers. Both α and β subunits have a large NH_2_-terminal extracellular domain, a single transmembrane domain and a short non-catalytic cytoplasmic tail [14, 15]. Integrins are involved in a wide range of biological activities and pathological processes, especially carcinogenesis and cancer progression [16, 17]. They regulate cell–cell and cell–ECM interactions, and this ‘outside-in’ signaling activates a number of signaling pathways that are important in the regulation of cell shape, survival, gene transcription and migration [18, 19]. They also interact with growth factors or chemokines to regulate cell growth and differentiation [14, 20]. Thus, targeting integrins and their associated signaling pathways, which are involved in tumor proliferation, migration, invasion and metastasis, may be a novel strategy for the diagnosis and treatment of cancers as well as certain non-neoplastic conditions [21]. Studies have also reported that effective blockage of integrins and ECM interactions impairs several important aspects of tumor biology and increases sensitivity to existing chemotherapy [22, 23]. Anti-integrin antagonists used in combination with current chemotherapeutic drugs have been shown to have roles in preventing drug resistance and tumor relapse [24].

In this study, we evaluated the prognostic predictive value of integrin family genes and *ITGA1* single nucleotide polymorphism (SNP) rs988574 in HBV-related HCC patients by performing prognostic analysis in 221 newly diagnosed pathologically confirmed HBV-related HCC patients. First, we found that *ITGA5* and *ITGB5* are more highly expressed in HBV-related HCC tissues than adjacent normal tissues. Conversely, *ITGA2B* is more highly expressed in adjacent normal tissues than in HBV-related HCC tissues. We also found that higher expression of *ITGA5*, *ITGB5* and *ITGA2B* predicted worse prognosis in HBV-related HCC patients, and patients with lower expression of both *ITGA5* and *ITGB5* had the best prognosis. In addition, we found that patients carrying the AG or GG genotypes at rs988574 had better prognosis than those with the AA genotype.

## RESULTS

### Patient characteristics and clinical predictors

The clinical and pathologic characteristics of the patients are shown in Table 1. Overall, 55 female patients and 430 male patients were included. Among them, 307 were Han Chinese and 178 were minorities. The median survival times were 57 and 51 months for patients aged ≤46 years and >46 years, respectively. As shown in Table 1 univariate analysis indicated that patients with BCLC stages B and C (HR = 1.92, 95%CI = 1.35–2.73; HR = 3.1, 95%CI = 2.31–4.16, respectively), Child-Pugh class B (HR = 1.68, 95%CI = 1.21–2.33), non-radical resection (HR = 0.76, 95%CI = 0.59–0.99), non-antiviral therapies (HR = 0.72, 95%CI = 0.53–0.98), AFP ≥ 300ng/ml (HR = 1.30, 95%CI = 0.99–1.71), tumor size ≥ 5 cm (HR = 2.04, 95%CI = 1.49–2.80), multiple tumors (n > 1) (HR = 1.61, 95%CI = 1.23–2.12) and presence of PVTT (HR = 2.40, 95%CI = 1.12–5.12) had higher risk of death when compared with patients with BCLC stage A, child-Pugh class A, radical resection, antiviral therapies, AFP < 300 ng/ml, tumor size < 3cm, single tumor (n = 1) and absence of PVTT, respectively. In addition, clinical features including age, gender, race, BMI, drinking status, smoking status, adjuvant TACE and cirrhosis were found to have no effect on the OS of HBV-related HCC patients in our study.

**Table 1:** Univariate Cox proportional hazards analysis of clinicopathological characteristics and overall survival in HBV-related HCC patients

Variables	Patients (n=485)	OS		
		MST (months)	HR * (95% CI)	P *
Age (yr)				
≤ 46	260	57	Ref.	
> 46	225	51	0.97 (0.74–1.25)	0.789
Gender				
male	430	51	Ref.	
female	55	80	0.75 (0.47–1.18)	0.208
Race				
Han	307	68	Ref.	
Minority	178	51	1.10 (0.84–1.45)	0.473
BMI				
≤ 25	401	58	Ref.	
> 25	84	57	0.95 (0.68–1.32)	0.737
Smoking status				
None	318	71	Ref.	
Ever	167	42	1.20 (0.91–1.57)	0.191
Drinking status				
None	295	71	Ref.	
Ever	190	48	1.18 (0.91–1.53)	0.219
Adjuvant TACE ^a^				
No	212	88	Ref.	
Yes	273	47	1.14 (0.87–1.49)	0.340
BCLC stage				
A	284	95	Ref.	**< 0.001**
B	80	47	1.92 (1.35–2.73)	**< 0.001**
C	121	24	3.10 (2.31–4.16)	**< 0.001**
Child–Pugh class				
A	403	65	Ref.	
B	82	34	1.68 (1.21–2.33)	**0.002**
Cirrhosis				
No	58	82	Ref.	
Yes	426	51	1.21 (0.80–1.84)	0.361
Antiviral therapy^b^				
No	315	47	Ref.	
Yes	170	81	0.72 (0.53–0.98)	**0.036**
				
AFP				
≤ 400 (ng/ml)	247	63	Ref.	
> 400 (ng/ml)	202	42	1.30 (0.99–1.71)	0.059
missing	36			
Radical resection				
No	205	41	Ref.	
Yes	268	74	0.76 (0.59–0.99)	**0.044**
missing	12			
Pathological grade				
Well	27	79	Ref.	0.761
Moderately	372	51	1.25 (0.68–2.30)	0.470
Poorly	13	NA	1.15 (0.40–3.31)	0.797
missing	73			
Oncological behavior				
Tumor size				
≤ 5 cm	155	123	Ref.	
> 5 cm	330	40	2.04 (1.49–2.80)	**< 0.001**
No. of tumors				
Single (n = 1)	356	63	Ref.	
Multiple (n > 1)	129	35	1.61 (1.23–2.12)	**0.001**
Regional invasion				
Absence	412	68	Ref.	
Presence	73	37	1.62 (1.137–2.30)	**0.007**
Intrahepatic metastasis				
Absence	264	81	Ref.	
Presence	221	35	1.80 (1.38–2.33)	**< 0.001**
Vascular invasion				
Absence	399	78	Ref.	
Presence	86	18	3.16 (2.35–4.25)	**< 0.001**
PVTT				
No	409	76	Ref.	**< 0.001**
vp1	11	28	2.40 (1.12–5.12)	**0.024**
vp2	17	17	3.44 (1.99–5.96)	**< 0.001**
vp3	40	17	2.89 (1.96–4.27)	**< 0.001**
vp4	8	8	5.84 (2.72–12.54)	**< 0.001**

Note: *HR and *P*-value for univariate survival analysis;^a^Adjuvant TACE post hepatectomy;^b^Adjuvant antiviral therapy post hepatectomy;

OS, overall survival; MST, median survival time; HR, hazard ratio; 95% CI, 95% confidence interval; Ref., reference; PVTT, portal vein tumor thrombus.

### *ITGA1* SNP rs988574-AA predicted worse OS in HBV-related HCC patients

**Figure 1:**
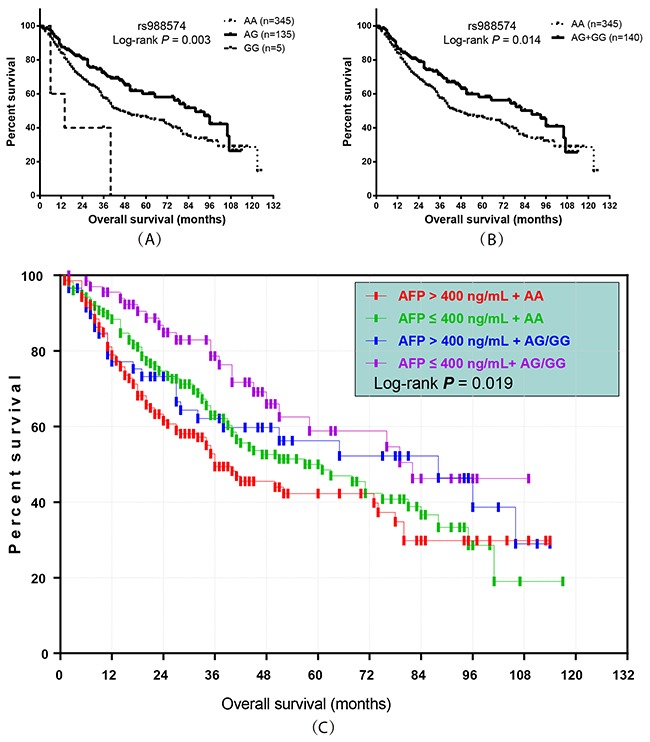
Prognostic value of *ITGA1* SNP rs988574 genotype in HBV-related HCC patients **(A, B)** Kaplan-Meier graphs representing the probabilities of overall survival in HCC patients; **(C)** Kaplan-Meier graph showing the overall survival by rs988574 genotype combined with serum AFP level in HBV-related HCC patients.

The SNP functional prediction results showed that rs988574 located in a splice site and the non-synonymous mutation was possibly damaging for gene expression as predicted by Polyphen in SNP selection tools. In this study, we found that patients carrying the AG/GG alleles of rs988574 had a significantly better prognosis when compared to patients with the AA genotype. Cox proportional hazards regression analysis showed that rs988574 was significantly associated with prognosis when adjusting for age, gender, race, smoking status, drinking status, BMI, child-Pugh class, cirrhosis, BCLC stage, pathological grade, TACE status post hepatectomy, antiviral therapy after hepatectomy, radical resection and serum AFP levels (Tables 2 and 3). Figure 1A shows that patients with the AG genotype had a significantly favorable OS, compared to patients with the AA genotype. Additionally, when combining carriers of the AG and GG alleles, patients with the AA genotype had a worse prognosis (Figure 1B). The adjusted survival curve shows a significant difference in OS between patients with the AG and GG genotypes of rs988574 compared to those with the AA genotype (*P* = 0.025, HR = 0.69 95%CI = 0.50–0.96; Table 3).

**Table 2:** Multivariate Cox proportional hazards ratio analysis of *ITGA1* SNPs and overall survival or relapse free survival in HBV-related HCC patients

SNP	Chr	Position	Gene	Allele	Function	MAF	os	
							Log-rank P	Cox P
rs1531545	5	52193287	ITGA1	C/T	Synonymous	0.34	0.208	0.301
rs4145748	5	52201722	ITGA1	C/T	Nonsynonymous	0.09	0.414	0.455
rs2279587	5	52214581	ITGA1	G/A	Nonsynonymous	0.11	0.257	0.312
rs12520591	5	52229745	ITGA1	T/G	Nonsynonymous	0.11	0.378	0.356
**rs988574**	**5**	**52240810**	**ITGA1**	**A/G**	**Nonsynonymous**	**0.14**	**0.002**	**0.043£**
rs2230392	17	48155425	ITGA3	G/A	Nonsynonymous	0.29	0.219	0.374
rs1143674	2	182374534	ITGA4	A/G	Synonymous	0.36	0.453	0.499
rs1143676	2	182395345	ITGA4	G/A	Nonsynonymous	0.12	0.182	0.386
rs7562325	2	182399097	ITGA4	T/C	Synonymous	0.36	0.420	0.429
rs11895564	2	173339808	ITGA6	G/A	Nonsynonymous	0.06	0.516	0.604
rs1800974	12	56089357	ITGA7	C/T	Nonsynonymous	0.06	0.108	0.156
rs2298033	10	15649710	ITGA8	G/A	Nonsynonymous	0.06	0.886	0.901
rs2507941	3	37536056	ITGA9	C/T	Synonymous	0.08	0.737	0.825
rs267561	3	37574951	ITGA9	G/A	Nonsynonymous	0.22	0.284	0.355
rs2274616	1	145536082	ITGA10	G/A	Nonsynonymous	0.07	0.479	0.622
rs2230433	16	30518041	ITGAL	G/C	Nonsynonymous	0.19	0.766	0.812
rs871443	17	73753503	ITGB4	T/C	Nonsynonymous	0.37	0.580	0.635
rs2291089	3	124515636	ITGB5	C/T	Nonsynonymous	0.10	0.299	0.427

Note: Cox P adjusted for age, gender, BMI, race, smoking status, drinking status, Child-Pugh class, cirrhosis, BCLC stage, pathological grade, TACE status post hepatectomy, antiviral therapy after hepatectomy, radical resection, serum AFP levels, intrahepatic metastasis, vascular invasion and PVTT. £Sequencing map of rs988574 was shown in Supplementary Figure 1.

SNP, single nucleotide polymorphism; Chr, chromosome; MAF, minor allele frequency; OS, overall survival.

**Table 3:** Multivariate Cox proportional hazards ratio analysis of *ITGA1* SNP rs988574 and overall survival in HBV-related HCC patients

SNP	Patients (n=485)	OS				
		MST (months)	HR (95% CI)	*P*	Adjusted HR* (95%CI)	Adjusted *P* *
rs988574						
AA	345	42	Ref.	**0.003**	Ref.	0.052
AG	135	88	0.65 (0.47–0.88)	**0.006**	0.67 (0.48–0.93)	**0.018**
GG	5	14	2.53 (0.94–6.83)	0.067	1.30 (0.40–4.25)	0.659
AG+GG	140	82	0.68 (0.51–0.93)	**0.014**	0.69 (0.50–0.96)	**0.025**

Note: *Adjusted for age, gender, BMI, race, smoking status, drinking status, Child-Pugh class, cirrhosis, BCLC stage, pathological grade, TACE status post hepatectomy, antiviral therapy after hepatectomy, radical resection, serum AFP levels, intrahepatic metastasis, vascular invasion and PVTT.

SNP, single nucleotide polymorphism; OS, overall survival; MST, median survival time; HR, hazard ratio; 95% CI, 95% confidence interval; Ref., reference.

**Figure 2:**
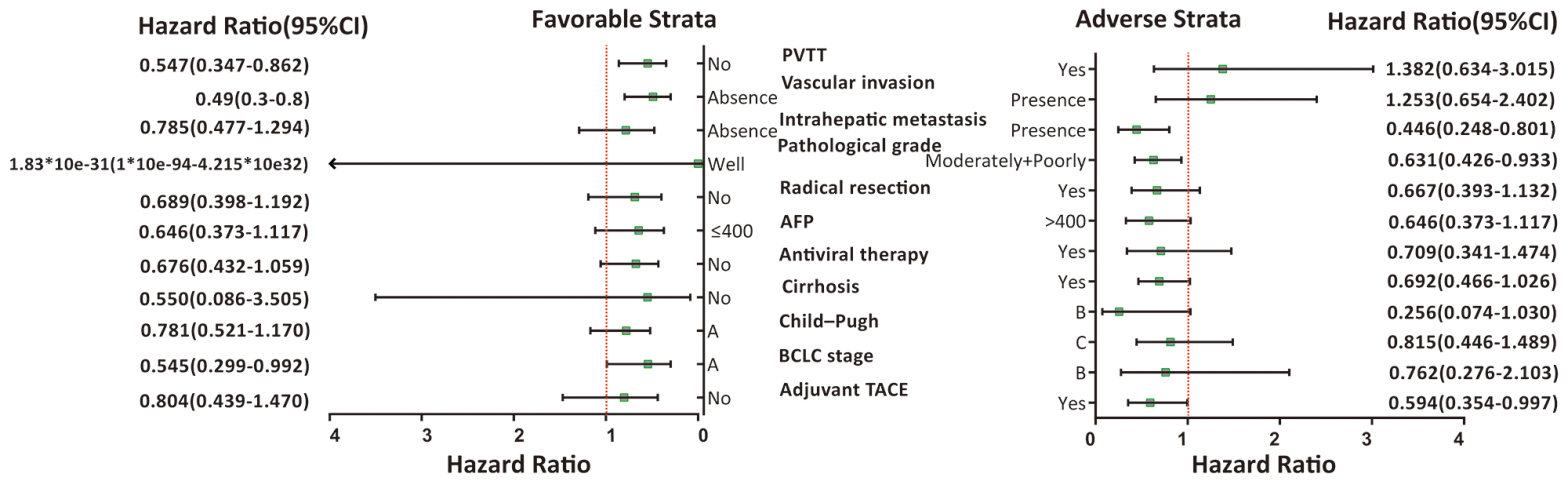
Stratified analysis of association between *ITGA1*-rs988574 polymorphisms and OS in HBV-related HCC patients Variables were stratified according to favorable and adverse strata.

Stratified analysis also utilized to investigate the association between clinicopathological features and HBV-related HCC outcomes. In stratified analyses, the AG/GG genotype ofrs988574 significantly decreased risk of death among HBV-related HCC patients with BCLC A stage, intrahepatic metastasis, advanced pathological grade, adjuvant TACE and patients without PVTT and vascular invasion (Figure 2) after hepatectomy, compared to patients with the AA genotype.

### Prognostic value of rs988574 combined with serum AFP level on OS in HBV-related HCC patients

In this study, we further analyzed the combined effect of rs988574 and serum AFP level on the prognosis of patients. According to rs988574 genotype and serum AFP level, patients were classified into four groups: AFP ≤ 400 ng/mL with AG/GG genotype, AFP ≤ 400 ng/mL with AA genotype, AFP > 400 ng/mL with AG/GG genotype and AFP > 400 ng/mL with AA genotype (Table 4). Multivariate Cox regression analysis indicated that, as compared to patients with AG/GG genotype and low serum AFP (AFP ≤ 400 ng/ml), patients with the AA genotype and a high serum AFP (AFP > 400 ng/mL) had a significantly higher risk for death (adjusted *P* = 0.026, adjusted HR = 1.75, 95%CI = 1.07–2.85; Table 4 and Figure 1C).

**Table 4:** Multivariate Cox proportional hazards ratio analysis combining *ITGA1* SNP rs988574 and APF level for overall survival in HBV-related HCC patients

SNP	MST (months)	HR (95%CI)	P	HR *(95%CI)	P *
rs988574					
AFP ≤ 400 (ng/mL) +AG/GG	79	Ref.	**0.019**	Ref.	0.094
AFP > 400 (ng/mL) + AG/GG	88	1.41 (0.80–2.48)	0.230	1.23 (0.68–2.22)	0.490
AFP ≤ 400 (ng/mL) + AA	57	1.59 (1.00–2.53)	**0.048**	1.60 (0.99–2.59)	0.056
AFP > 400 (ng/mL) + AA	36	2.05 (1.28–3.27)	**0.003**	1.75 (1.07–2.85)	**0.026**

Note:*Adjusted for age, gender, BMI, race, smoking status, drinking status, Child-Pugh class, cirrhosis, BCLC stage, pathological grade, TACE status post hepatectomy, antiviral therapy after hepatectomy, radical resection, intrahepatic metastasis, vascular invasion and PVTT.

SNP, single nucleotide polymorphism; MST, median survival time; HR, hazard ratio; 95% CI, 95% confidence interval; Ref., reference.

### The mRNA expression level of integrins in HCC tissues and adjacent normal tissues

**Figure 3:**
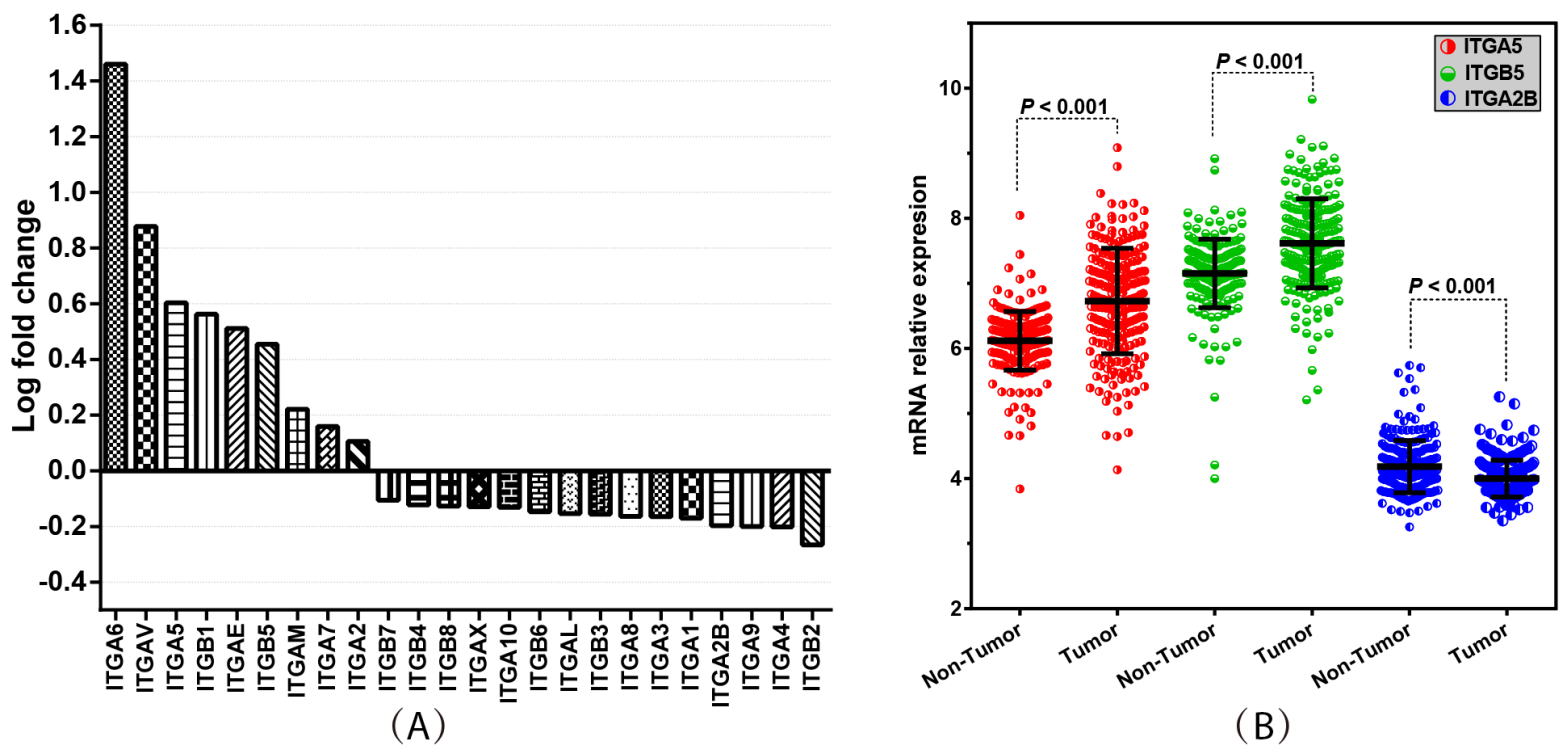
Integrin family genes’ expression levels in HCC tissues and adjacent normal tissues **(A)** Histogram of log-fold change showing the 24 integrin family members’ expression levels in HBV-related HCC tissues compared with adjacent normal tissues. **(B)** Comparison of the expression of *ITGA5*, *ITGB5* and *ITGA2B* in 221 HBV-related HCC tissues and adjacent normal tissues. *P-*value indicates statistical significance analyzed by Student's t-test.

The integrin family consists of 24 genes, widely studied in the progression of various cancers. To clarify the expression pattern of integrin family members in HBV-related HCC patients, we compared the mRNA expression levels between HBV-related HCC tissues and adjacent normal tissues. The results showed that nine of the integrin gene family members have a higher expression in HBV-related HCC tissues compared to adjacent normal tissues (Figure 3A; *P* < 0.001). In a multivariate Cox proportional hazards ratio analysis adjusting for age, gender, cirrhosis, BCLC stage and serum AFP levels, we found that among the 24 integrin family genes, *ITGA5*, *ITGB5* and *ITGA2B* were significantly associated with prognosis in terms of OS or RFS of patients with HBV-related HCC (Tables 5 and 6; *P* < 0.05). Then, we compared the expression of *ITGA5*, *ITGB5* and *ITGA2B* in 221 HBV-related HCC tissues and 221 adjacent normal tissues, and the results showed that *ITGA5* and *ITGB5* are more highly expressed in HBV-related HCC tissues than adjacent normal tissues, while *ITGA2B* had a lower expression level in HBV-related HCC tissues than adjacent normal tissues (Figure 3B; *P* < 0.001).

**Table 5:** Multivariate Cox proportional hazards ratio analysis of integrin genes expression and overall survival or relapse free survival in HBV-related HCC patients

Integrins	OS		RFS	
**Log-rank P**	Cox P*	**Log-rank P**	Cox P*
ITGA1	0.861	0.438	0.793	0.471
ITGA2	0.241	0.762	0.305	0.727
**ITGA2B**	0.230	**0.064**	0.172	**0.032**
ITGA3	0.209	0.217	0.721	0.997
ITGA4	0.138	0.327	0.069	0.095
**ITGA5**	**0.013**	**0.041**	**0.003**	**0.006**
ITGA6	0.885	0.337	0.377	0.089
ITGA7	0.126	0.169	0.438	0.407
ITGA8	0.579	0.826	0.877	0.784
ITGA9	0.481	0.813	0.094	0.072
ITGA10	0.509	0.567	0.308	0.510
ITGAE	0.528	0.464	0.906	0.913
ITGAL	0.803	0.913	0.562	0.672
ITGAM	0.463	0.677	0.439	0.777
ITGAV	0.411	0.172	0.825	0.692
ITGAX	0.877	0.079	0.517	0.879
ITGB1	0.403	0.822	0.340	0.846
ITGB2	0.902	0.654	0.514	0.298
ITGB3	0.614	0.154	0.042	0.064
ITGB4	0.716	0.983	0.735	0.407
ITGB5	**0.016**	**0.048**	0.069	**0.042**
ITGB6	0.434	0.928	0.625	0.768
ITGB7	0.451	0.635	0.523	0.591
ITGB8	0.945	0.919	0.615	0.760

Note: The 75th percentile of mRNA expression in the total population was used as the cutoff point to define lower and higher expression groups. *Cox P adjusted for age, gender, cirrhosis, BCLC stage and serum AFP levels.

OS, overall survival; RFS, relapse-free survival.

**Table 6:** Multivariate Cox proportional hazards ratio analysis of *ITGA5*, *ITGB5* and *ITGA2B* expression and overall survival or relapse-free survival in HBV-related HCC patients

Gene	Patients (n=221)	OS			RFS		
**MST (months)**	HR* (95%CI)	P value*	**MRT (months)**	HR* (95%CI)	P value*
ITGA5							
Lower	165	67.4	Ref.	**0.041**	51.6	Ref.	**0.006**
Higher	56	45.9	1.64 (1.02–2.63)		21.3	1.75 (1.18–2.62)	
ITGB5							
Lower	165	67.4	Ref.	**0.048**	49.1	Ref.	**0.042**
Higher	56	53	1.62 (1.00–2.60)		23.6	1.53 (1.02–2.31)	
ITGA2B							
Lower	165	67.3	Ref.	0.064	48	Ref.	**0.032**
Higher	56	58.4	1.58 (0.97–2.73)		24.6	1.56 (1.04–2.33)	

Note: The 75th percentile of mRNA expression in the total population was used as the cutoff point to define lower and higher expression groups. *Adjusted for age, gender, cirrhosis, BCLC stage and serum AFP levels.

OS, overall survival; RFS, relapse-free survival; MST, median survival time; MRT, median relapse time; HR, hazard ratio; 95% CI, 95% confidence interval; Ref., reference.

**Figure 4:**
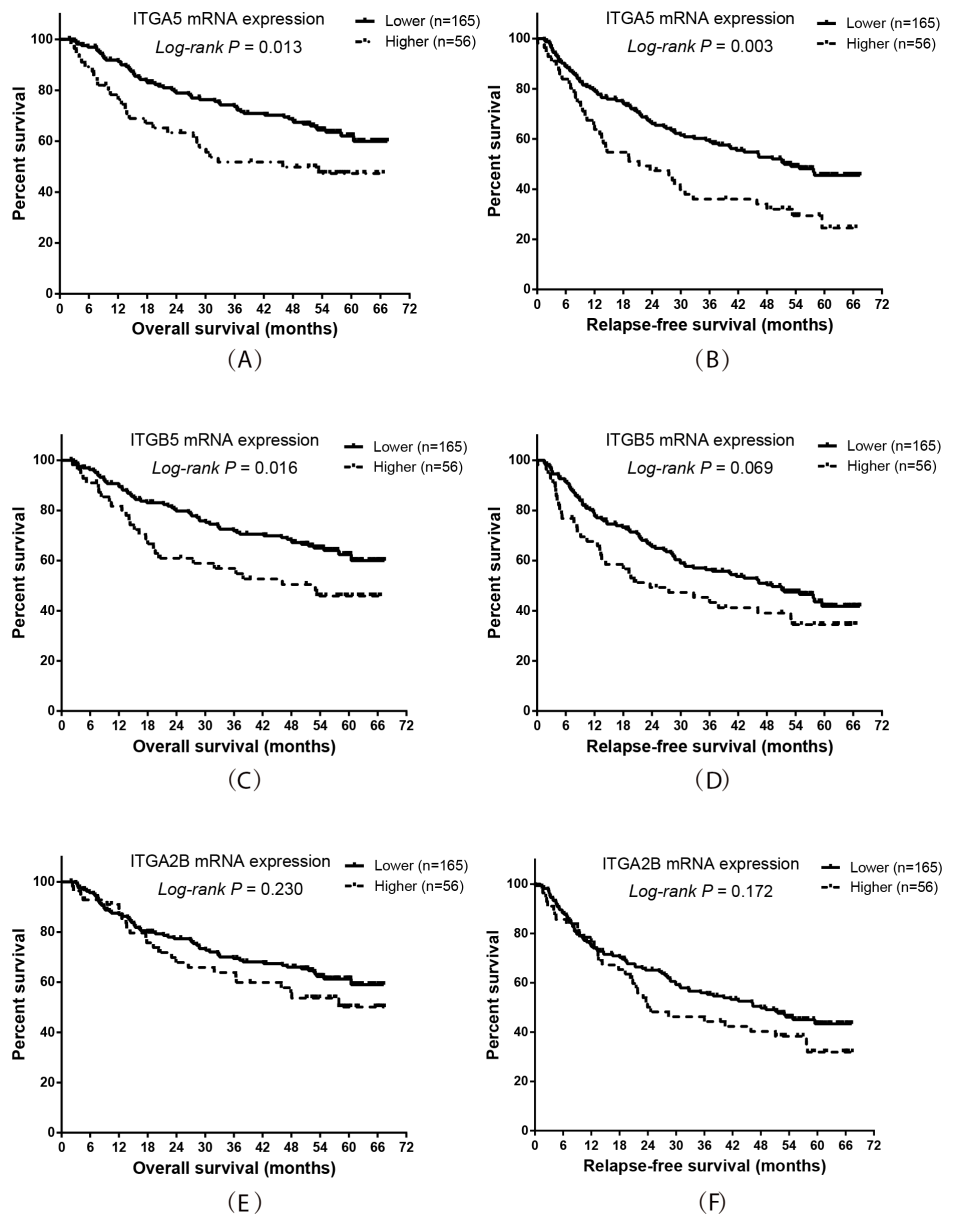
Prognostic value of *ITGA5*, *ITGB5* and *ITGA2B* expression levels in HBV-related HCC patients Kaplan-Meier plot representing the probabilities of overall survival and relapse-free survival in HCC patients stratified according to the expression levels of *ITGA5* **(A, B)**, *ITGB5* **(C, D)** and *ITGA2B* **(E, F)**.

The adjusted survival curves showed a significant difference in OS and RFS between patients with higher versus lower *ITGA5*, *ITGB5* and *ITGA2B* expression levels. (Figure 4). The mRNA expression levels of *ITGA5*, *ITGB5* and *ITGA2B* were independent prognostic indicators in HBV-related HCC patients (Table 6). Co-expression analysis indicated that *ITGA5* mRNA expression had a significantly positive correlation with *ITGB5* (r=0.32, *P<*0.001) in tumor tissue, whereas *ITGA2B* had a significantly negative correlation with *ITGA5* (r=-0.142, *P* =0.035) and *ITGB5* (r=-0.34, *P* <0.001), respectively.

### Prognostic prediction of *ITGA5* expression combined with *ITGB5* expression in HBV-related HCC patients

**Figure 5:**
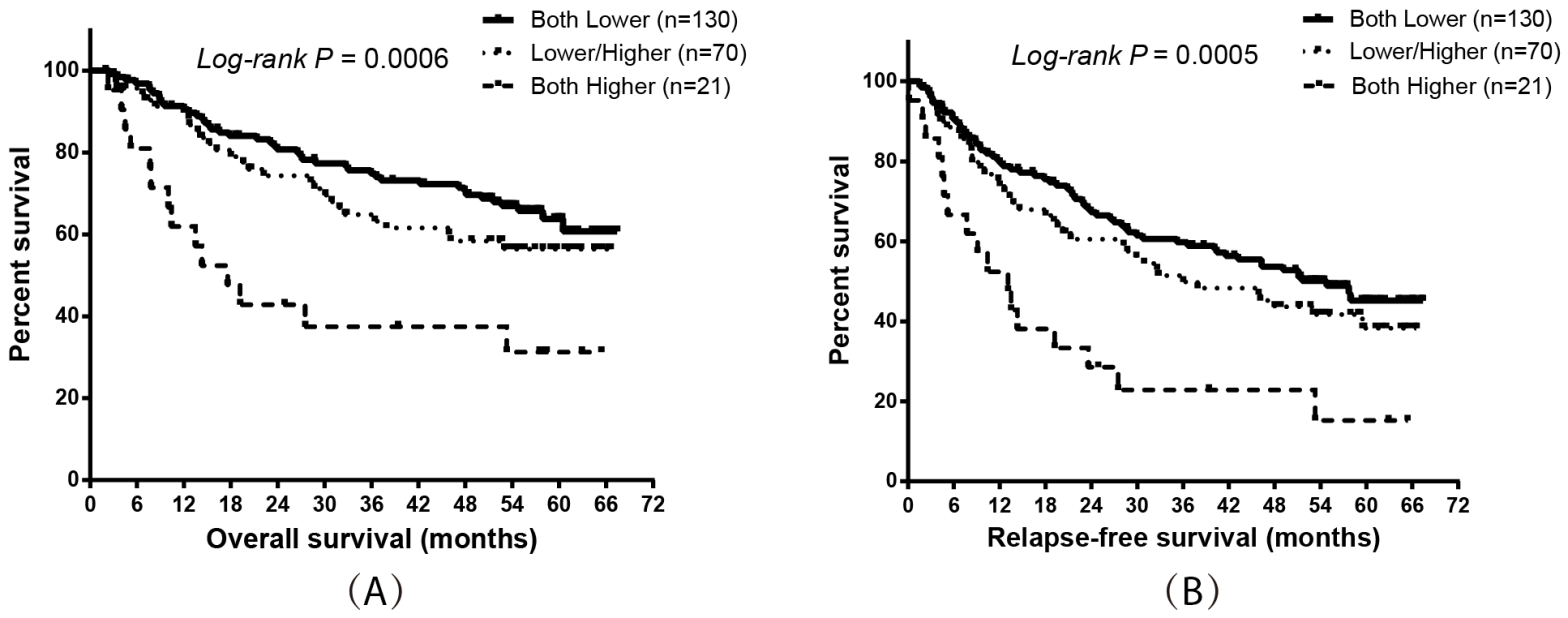
Prognostic value of *ITGA5* expression combined with *ITGB5* expression in HBV-related HCC patients Kaplan-Meier plots showing the overall survival **(A)** and relapse-free survival **(B)** of combining *ITGA5* and *ITGB5* mRNA expression.

Base on the results of the co-expression and survival analysis for individual integrin genes, we analyzed the prognostic predictive value of combining *ITGA5* and *ITGB5* expression. The results showed that patients with higher expression of both *ITGA5* and *ITGB5* had the worst OS and RFS compared to patients with lower expression of these genes (Figure 5 and Table 7; *P =* 0.017 and 0.002, respectively).

**Table 7:** Multivariate Cox proportional hazards ratio analysis combining *ITGA5* and *ITGB5* expression and overall survival or relapse-free survival in HBV-related HCC patients

Groups (ITTGA5 & ITGB5)	Patients (n=221)	OS			RFS		
**MST (months)**	HR* (95%CI)	P value*	**MRT (months)**	HR* (95%CI)	P value*
Both Lower	130	67.4	Ref.	**0.017**	54.8	Ref.	**0.002**
Lower/Higher	70	53.3	1.37 (0.84–2.25)	0.212	36.0	1.38 (0.91–2.07)	0.127
Both Higher	21	17.6	2.58 (1.34–4.95)	**0.004**	13.1	2.89 (1.61–5.18)	**<0.001**

Note: The 75th percentile of mRNA expression in the total population was used as the cutoff point to define lower and higher expression groups. *Adjusted for age, gender, cirrhosis, BCLC stage and serum AFP levels.

OS, overall survival; RFS, relapse-free survival; MST, median survival time; MRT, median relapse time; HR, hazard ratio; 95% CI, 95% confidence interval; Ref., reference.

## DISCUSSION

In this study, we examined whether genetic polymorphisms in the integrin gene family are associated with the OS of HBV-related HCC patients and explored the expression pattern and prognostic predictive value of these genes using GEO data. We found that the AG/GG genotypes of rs988574 (*ITGA1*) are significantly associated with better OS compared with the AA genotype in HBV-related HCC patients. In addition, we found that patients with the AA genotype combined with high serum AFP (AFP > 400 ng/mL) predicted the highest risk of death. Additionally, among the 24 integrin family members, nine had higher expression in HBV-related HCC tissues compared to adjacent normal tissues, while the other integrin genes showed the opposite expression pattern. Specifically, the expression of *ITGA5* and *ITGB5* were higher in HCC tissues than in adjacent tissues. In contrast, the expression of *ITGA2B* was the opposite, and significantly negative correlation with *ITGA5* and *ITGB5* in tumor tissue. Furthermore, we found that the expression level of *ITGA5*, *ITGB5* and *ITGA2B* were associated with the prognosis of HBV-related HCC patients; lower expression of these genes predicted a better OS and RFS compared with higher expression. Furthermore, we found that patients with the combination of lower expression of both *ITGA5* and *ITGB5* had the best OS and RFS.

Our SNP functional prediction results show that rs988574 disrupts a splice site, and this non-synonymous mutation was possibly damaging for gene expression as predicted by Polyphen in SNP selection tools. This may a potential mechanism that AA polymorphism of rs988574 affects functionality of *ITGA1* in respect to the HBV-related HCC. Moreover, we demonstrated that the SNP rs988574 in *ITGA1* may serve as an independent prognostic marker in HBV-related HCC patients. Once validated, these genes, either alone or in combination with each other or other traditional clinical-prognostic factors, may be used as new targets for the treatment of HBV-related HCC patients.

Integrins are a large family of cell surface receptors that bind ECM proteins to regulate attachment, cell proliferation, differentiation, motility and other essential cell functions [25]. They are critically important for both health and disease by participating in diverse human pathologies including thrombotic diseases, infectious diseases, inflammation, fibrosis and cancer [24]. Integrins play an important role in cancer survival, proliferation, growth and metastasis [26–28]. Apart from their correlation with cancer prognosis, integrins can also protect cells from stress, leading to cancer cell resistance to radio- and chemotherapy [29]. Additionally, integrin inhibition has been shown to enhance the cytotoxic efficacy of radiation and chemotherapeutics [30]. Reports have also shown that integrins are sensitive to pharmacological blockade, making them exciting pharmacological targets for anticancer therapies [31]. Indeed, several integrin inhibitors have been designed and have undergone clinical trials, and all were shown to be nontoxic.

Some studies have shown that integrins, such as beta1, beta3 and beta5, play an important role in cell growth, proliferation, invasion and migration [31]. It has also been demonstrated that integrin levels are frequently elevated in aggressive tumors, implying that these proteins might be promising targets for cancer treatments [32]. ITGA5 is a member of the integrin family that mediates cell-to-cell adhesion, migration and anoikis in virous tumors both *in vivo* and *in vitro* [33, 34]. Additionally, ITGA5 promotes tumor cell adhesion and migration through activating focal adhesion kinase (FAK), and an antibody (Volociximab) or a non–RGD-based peptide inhibitor (ATN-161), which blocks the function of the heterodimer function, significantly inhibits the growth and metastasis of breast cancer cells [23, 34]. ITGB5 also promotes intracellular signaling by recruiting and activating integrin-associated kinases, including FAK, which contributes to chemoresistance in malignant disease. FAK, interacting with Src at Tyr861, plays a vital role in ITGB5-mediated signaling in response to vascular endothelial growth factor (VEGF) and Ras transformation in fibroblasts [31]. These features suggest that ITGB5 may participate in tumor cell adhesion, migration, inhibits metastasis, and angiogenesis, all of which may influence tumor prognosis. Our current study demonstrated that ITGB5 expression was related to HBV-related HCC prognosis, and the features of ITGB5 may play a role in HBV-related HCC prognosis, but this hypothesis still need further functional experiment investigation. ITGA2B, also known as platelet glycoprotein IIb of the IIb/IIIa complex, is the most abundant receptor on the platelet surface [35]. By binding platelets together, it has a significant role in hemostasis. Recent studies have suggested that platelets may contribute to the spread of cancer, and cancer patients may have both an elevated number of, and activated, platelets [36]. One study also reported that the platelet glycoprotein IIb/IIIa (GP IIb/IIIa) is crucial for the hematogenous metastasis of human breast carcinoma cells [37]. Based on previous research, these functions may play a role in the prognostic of ITGA2B expression in HBV-related HCC.

In previous reports, focus has been placed on investigating the biological behavior and prognostic roles of integrin family members in various cancers. Our results were consistent with a previous study, which found that the expression of *ITGA5* was associated with the prognosis of HBV-related HCC patients [34]. Furthermore, we found that the *ITGB5* expression level was higher in HCC tissues, while *ITGA2B* was higher in adjacent normal tissues. Furthermore, both of these genes were associated with the prognosis of HBV-related HCC patients. In addition, this is the first study to detect the prognostic predictive value of combining the expression of *ITGA5* with *ITGB5*. Finally, we found that the AG/GG genotypes at rs988574 (*ITGA1*) are significantly associated with better OS compared with carriers of the AA genotype in HBV-related HCC patients. The biggest challenge in this study was how to detect gene expression homogeneously since the data was influenced by various conditions, such as bias from the specific cell populations in the tumor tissues, the sampling sites and sample degradation, problems that have also occurred in other studies.

In summary, our study demonstrated that the expression of *ITGA5*, *ITGB5* and *ITGA2B*, as well as the genotype at rs988574 (*ITGA1*), may be potential independent prognostic bio-markers and therapeutic targets for HBV-related HCC patients and may be useful in the diagnosis of HBV-related HCC patients. Further studies focused on integrin family members and their downstream signaling pathways, as well as the effective blockage of integrins and their ECM interactions, will provide additional information about their use in cancer therapies.

## METERIALS AND METHODS

### Ethical approval

This study was performed in support of the Ethical Review Committee of the First Affiliated Hospital of Guangxi Medical University (Guangxi, China), and informed consent was obtained from all patients.

### Study population

A total of 485 newly diagnosed pathologically confirmed HBV-related HCC patients that had undergone surgical resection were recruited at the First Affiliated Hospital of Guangxi Medical University (Guangxi, China) from January 2005 to September 2014. All the patients were diagnosed by histopathological examination which followed the National Comprehensive Cancer Network (NCCN) clinical practice guidelines for oncology. The patients were followed up via telephone or hospital visit until death or final follow-up in September 2014, for a median follow-up time of 47 months. None of the patients had a previous cancer diagnosis of any kind at the initial screening examination. The clinicopathological characteristics of patients including age, gender, smoking status, drinking status, pathological grade, biobehavior of their cancer, serum AFP level, hepatic cirrhosis, radical resection and use of transcatheter hepatic arterial chemoembolization (TACE) were obtained from medical records and pathological reports. Tumor status was classified according to the BCLC staging system. Child-Pugh class was defined as previously published. Portal vein tumor thrombus (PVTT) was determined to be absent or present. The endpoint was overall survival (OS), which was calculated from the date of pathological diagnosis/recruitment to death or the end of follow-up.

### SNP selection, DNA extraction and genotyping

SNP selection tools (http://snpinfo.niehs.nih.gov/snpinfo/snpfunc.htm) were used to select candidate SNPs in integrin genes according a previous publication. The inclusion criteria were as follows: (i) SNPs in splice sites and CDS regions resulting in amino acid changes; SNPs in transcription factor binding sites of the 5’ flanking region; and SNPs in miRNA binding sites of 3’ UTR; and (ii) SNPs with minor allele frequency (MAF) ≥ 10% in the Han Chinese population (CHB) from the 1000 Genomes Project (March 2012, build GRCh37/hg19) [38]. These criteria resulted in18 SNPs being identified for assessment in this study (Table 8).

**Table 8:** Primers for SNPs of integrin genes

SNPs	Primers	Sequences (5′-3′)	Annealing temperature (°C)	Amp Length (bp)
**rs1531545**	ForwardReverse	ACAAGCTCTACCAGGTATCTGTATTCTTTCAAGCTATGAACTGCACGTTG	60	274
**rs4145748**	ForwardReverse	AGAGCATATTAAAAGCTTCCACCAACACCCATCCAACATGAAGACAAAG	62	509
**rs2279587**	ForwardReverse	GAAATGGAGTCCTGAGCGCTGTGGAGTGTTGAGGGCACTGCAT	62	533
**rs12520591**	ForwardReverse	CGTTTCAAGGTAAATGAATTCCCCTATGAGGCTTAGATCTCTAAACTGATGTG	61	510
**rs988574**	ForwardReverse	GAGGGAGGACAAGTGCACGGGCAATACCAGTCACTGCTTAAGGGA	62	376
**rs2230392**	ForwardReverse	CCCTCACCCAGAATAGGAGGAGGCCTCCATTTCTGCCTGCTTTACA	63	580
**rs1143674**	ForwardReverse	ATAGTGTTTGGCCCTTTTCAGGAATCTTCACTGGCAAGGCATTAAAA	61	508
**rs1143676**	ForwardReverse	TTGGCTGGGTTTTTGTGTTTCTGGACCCTGGGTCTATCTCAACTT	62	494
**rs7562325**	ForwardReverse	AAACCTCTAGCTAGAAGGTAAAGATCCGCCCATTTTCTTGCTGGTTCTAATA	62	515
**rs11895564**	ForwardReverse	GGAGTCCTGCTGTACTATGGTTCTCAGCAGCGCTATTATTTAAACAATCA	61	501
**rs1800974**	ForwardReverse	GTCCTCTTCCACCTTCTGCCTTTCAGTGTGGCCCAGCTCTTGAC	61	493
**rs2298033**	ForwardReverse	AATGGTTGGAATTTGGATATGAAGGAGATCTGATAGAGCACTGTGTTCACTG	60	272
**rs2507941**	ForwardReverse	GCACTCGTGGGAAGTGGCTGGGGCACAACCTGACTGAATGTC	60	501
**rs267561**	ForwardReverse	ATCCTGTTGGTCTAGTTCTTGTTTGTGGTCCAATGAGGCTTCAGTCTAG	62	512
**rs2274616**	ForwardReverse	GGGAAGCAAACACTGGGCTTGCTCCTTACCAGCACATGGAAGT	62	492
**rs2230433**	ForwardReverse	ATTTATTTCTTTCTGGCCCACCATAAACCTGGTACCTCGGATCATACA	60	410
**rs871443**	ForwardReverse	TAGGCACCTGTCCTTTCCTTCACCGAGTCGGGAGGACGCCTAGTG	62	497
**rs2291089**	ForwardReverse	AGCCGCACGTGCAGTTGTAGGGCTGGGAAGAGGATAGGACAGAA	62	494

SNPs, single nucleotide polymorphisms.

HCC tissues collected after surgical resection from HBV-related HCC patients were immediately stored at −80°C until DNA extraction using the TIANamp Genomic DNA Kit (Tiangen Biotech, Beijing, China), and genotyping was performed using Sanger DNA sequencingby Shanghai Sangon Biological Engineering Technology & Services (Shanghai, China). All primers are shown in Table 8.

### Integrin gene mRNA expression analyses in HBV-related HCC tissues

In the present study, we focus on the Chinese HBV-related HCC. The Gene Expression Omnibus (GEO) data selection criteria were set as follows: (i) expression profiling chip; (ii) Chinese HBV-related HCC; (iii) corresponding survival profiles available; (iv) patients undergoing hepatectomy. By searching the GEO database, we found that only the data of GSE14520 met the above criteria. Data were analyzed with Expression Console software (http://www.affymetrix.com/estore/index.jsp). Probe signal values were converted to log2 values, and annotated genes were analyzed using the corresponding Affymetrix HT Human Genome U133A and Human Genome U133A_2 array annotation files. A multi-array average algorithm was used for normalization of the GSE14520 mRNA expression data. We analyzed the association between the mRNA expression of integrin genes and prognosis in 221 HBV-related HCC patients using data from the GSE14520. The 75th percentile was used to define higher versus lower expression.

### Statistical analysis

The pearson correlation coefficient was used to assess the co-expression correlation of integrin genes in tumor mRNA. OS was defined as the time from surgery to death due to HCC and relapse-free survival (RFS) was defined as the time from surgery to disease recurrence. Hazard ratios (HRs) and 95% confidence intervals (CIs) were estimated by the Cox proportional hazards model after adjusting for factors such as age, gender, AFP level, tumor differentiation and treatment after surgery. GraphPad Prism 6 was used to plot Kaplan–Meier survival curves, scatter diagrams and histograms. Differences in the mRNA expression of integrin genes between HCC tissues and non-HCC tissues were evaluated using Student's t-test. Statistical significance was set at a level of *P* = 0.05, and all analyses were done using SPSS version 18.0 (SPSS, Inc., Chicago, IL, USA).

## SUPPLEMENTARY MATERIALS FIGURE



## Abbreviations

HCC: hepatocellular carcinoma
SNPs: single nucleotide polymorphisms
PCR: polymerase chain reaction
HBV: hepatitis B virus
SNPs: single nucleotide polymorphisms
OS: overall survival
RFS: recurrence-free survival
AFP: serum alpha fetoprotein
PVTT: portal vein tumor thrombosis
MAF: minor allele frequency
CDS: coding sequence
GEO: Gene Expression Omnibus
HR: hazard ratios
95% CI: 95% confidence interval
MST: median survival time
MRT: median recurrence time.
