# Supplementary material for: Prognostic value of integrin variants and expression in post-operative patients with HBV-related hepatocellular carcinoma
Source: Oncotarget. 2017 Aug 10;8(44):76816–31. doi: 10.18632/oncotarget.20161 (PMC5652745; doi:10.18632/oncotarget.20161)
Supplement: Supplementary file 1 [file oncotarget-08-76816-s001.pdf]

Prognostic value of integrin variants and expression in post-operative patients with HBV-related hepatocellular carcinoma

SUPPLEMENTARY MATERIALS

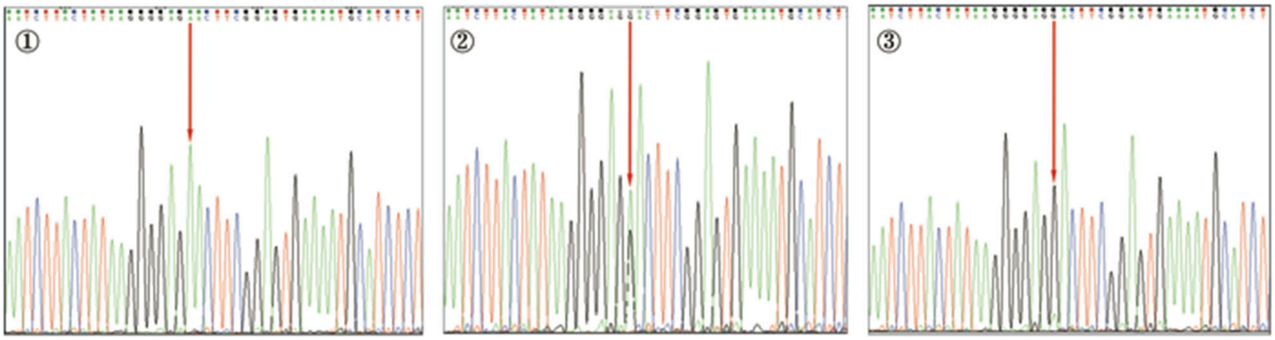

Supplementary Figure 1: Sequencing map for genotypes of *ITGA1*-rs988574 polymorphism. The arrows in ①–③ show AA, AG, and GG genotypes, respectively.
